# Non-aneurysmal Subarachnoid Hemorrhage Following Routine Dental Extraction: A Case Report

**DOI:** 10.7759/cureus.117936

**Published:** 2026-10-14

**Authors:** Yousif Alomar, Anas Adam, Umme Nashrah, Umm E Amara, Nissar Shaikh

**Affiliations:** 1 Medical Education, Hamad General Hospital, Doha, QAT; 2 Anesthesiology, Hamad General Hospital, Doha, QAT; 3 Medicine, Deccan College of Medical Sciences, Hyderabad, IND; 4 Obstetrics and Gynecology, Deccan College of Medical Sciences, Hyderabad, IND; 5 Surgical Intensive Care, Hamad General Hospital, Doha, QAT

**Keywords:** case report, external ventricular drain, hydrocephalus, subarachnoid hemorrhage, tooth extraction

## Abstract

We present the case of a 65-year-old man with multiple chronic comorbidities who developed severe headache and confusion three days after a routine dental extraction and was found to have a non-aneurysmal subarachnoid hemorrhage (SAH). On initial imaging, he had a diffuse SAH, and the ventricles were dilated, but no aneurysms or vascular malformations were identified on angiography. This was managed with the insertion of a right frontal external ventricular drain (EVD) to divert cerebrospinal fluid (CSF), with close monitoring in the intensive care unit (ICU).

The patient experienced gradual neurological and clinical improvement during a 12-day stay at the hospital, and fluid balance, electrolytes, pain management, and complications, including pneumocephalus, were carefully managed. The EVD was successfully weaned and removed once neurological status remained stable and repeat imaging was reassuring.

The patient was thereafter transferred to the neurosurgical ward for regular monitoring and repeat imaging, followed by discharge with outpatient follow-up. This case highlights the importance of multidisciplinary care and close monitoring in managing non-aneurysmal SAH with hydrocephalus in patients with complex medical histories.

## Introduction

One of the most widely used dental procedures is dental extraction to alleviate pain, treat infected teeth, or manage dental health complications [1]. As a controlled procedure, it is safe but has risks of bleeding, infection, nerve injury [2], and, in very rare situations, major neurological issues [3]. Subarachnoid hemorrhage (SAH) is a potentially life-threatening medical condition that is defined as bleeding into the subarachnoid space, usually due to the rupture of an intracranial aneurysm [4], although in some cases, it can occur without any identifiable vascular malformation - a so-called non-aneurysmal SAH [5].

In the systematic review by Shafa et al. [3], which pooled 47 patients from 42 studies reporting neurosurgical complications after tooth extraction, brain abscess and meningitis predominated, and only one case of SAH was identified; this was a fatal event reported in the review. Proposed mechanisms include pain- or stress-induced hemodynamic changes and the vasoconstrictor content of local anesthetics, and, more speculatively, transient rises in intracranial venous pressure from Valsalva-type straining, breath-holding, or coughing during the procedure, which commonly accompany the bracing and anxiety associated with extraction [6].

Although rare, these cases bring out the necessity of clinical awareness, particularly among patients who have preexisting vascular risk factors or comorbidities [7].

We report an extremely rare case of non-aneurysmal SAH with acute hydrocephalus following an uncomplicated dental extraction, highlighting the interplay between dental procedural stress and underlying cerebrovascular vulnerability.

## Case presentation

A 65-year-old male with a known history of hypertension, chronic kidney disease, type 2 diabetes, ischemic heart disease, dyslipidemia, and subclinical hypothyroidism was found in a confused, disoriented state next to his car and was subsequently admitted to the hospital. He did not report an episode of loss of consciousness, vomiting, seizures, or head trauma but stated that he had a severe headache, which started three days after a tooth extraction. His vital signs were stable at the time of arrival at the Emergency Department. His Glasgow Coma Scale (GCS) was 14 on admission. Computed tomography (CT) of the head indicated diffuse SAH with hydrocephalus, and an additional digital subtraction cerebral angiography performed on the day of admission indicated a lack of aneurysmal or vascular malformation, thus diagnosing the condition as non-aneurysmal SAH. In order to address the hydrocephalus, an external ventricular drain (EVD) was placed in the right frontal region, and the patient was shifted to the intensive care unit (ICU), where attention was given to his neurological status and supportive management was provided.

On Day 1, after undergoing an angiogram, the patient was admitted to the ICU with impaired consciousness, thus hindering neurological evaluation. Nevertheless, he had stable vital signs, was breathing spontaneously, and had reactive pupils. The EVD resulted in little cerebrospinal fluid (CSF). In the laboratory tests, there was continued hyperglycemia, a sodium level of 139, mild renal dysfunction, leukocytosis and neutrophilia, and metabolic acidosis. CSF was bloody in appearance and had increased red blood cells, raising strong suspicion for SAH in the absence of clinical or laboratory signs of active infection. The patient received intravenous fluids for volume management and insulin for glycemic control. Additionally, empiric antibiotic coverage with cefazolin, levetiracetam for seizure prophylaxis, and nimodipine for vasospasm prevention were initiated. Supportive care included analgesics, antiemetics, senna for bowel management, and electrolyte replacement. The primary controlled variable was blood pressure, with systolic blood pressure limited to less than 160 mmHg. The patient was assessed and placed on mechanical deep vein thrombosis (DVT) prophylaxis, with a decision regarding chemical prophylaxis. Enteral nutrition was initiated via a nasogastric tube, while antiplatelet and antihypertensive medications were withheld pending a complete cardiology evaluation. Monitoring was performed continuously according to the SAH protocol at the institution.

On Day 2, the patient demonstrated significant clinical improvement: he was fully alert with a GCS score of 15, hemodynamically stable, and breathing spontaneously on room air. The EVD was placed at a lower reading to allow more CSF drainage, which successfully improved CSF output alongside improving serum sodium levels. The patient tolerated the nasogastric tube well for nutritional support, so the decision was made by the care team to advance his diet and withdraw the inessential supportive lines. Pharmacologic DVT prophylaxis was withheld pending clearance by the neurosurgical team, though mechanical prophylaxis was maintained. The main clinical objectives were aimed at keeping the patient neurologically stable, achieving optimal fluid balance, and avoiding the risk of possible complications.

On Day 3, the patient remained clinically and neurologically stable at a GCS score of 15, without evidence of new neurological deficits. He was hemodynamically stable, was able to tolerate his renal diet, and showed positive fluid balance. His EVD was draining regularly, and his blood glucose levels were effectively controlled with his current insulin regimen. The laboratory results were normal, and pain was treated with analgesics. Supportive measures were continued, and arrangements were made to continue with repeat CT brain imaging to help monitor any changes or complications.

On Day 4, the patient was clinically and neurologically stable despite new imaging changes. Repeat CT demonstrated enhancement of hydrocephalus, reduction of ventricular size, and clearing of most of the previous intracranial blood. New findings, however, were minor pneumocephalus (air pockets in the brain), indistinct new SAH in the occipital area, blood traces in the right lateral ventricles, and air under the scalp in the area of EVD insertion. This was met with the administration of pharmacologic DVT prophylaxis, along with sodium supplementation. The treatment strategy focused on maintaining fluid and electrolyte balance through close monitoring.

On Day 5, the patient remained clinically stable and alert, breathing spontaneously on room air, with well-controlled pain and no focal neurological deficits. The EVD maintained output, and electrolytes were within normal limits. Supportive measures, including glycemic control and nutritional support, remained constant, aiding in the continued stability of the patient.

On Day 6, the patient was alert, neurologically intact, and mobilizing well, with overall clinical improvement noted since the previous day. Renal function, serum electrolytes, and laboratory parameters were normal. The EVD was raised to 15 cm H₂O for gradual CSF drainage weaning. Improved pain control allowed for the discontinuation of intravenous fentanyl patient-controlled analgesia (PCA). Close neurological monitoring and supportive care continued to be an important part of the plan.

On Day 7, the patient was stable and continued to recover, reporting only a mild occipital headache without any new symptoms. The EVD was maintained at 15 cm H₂O with regular flow of CSF. Follow-up CT revealed decreasing intracranial air (pneumocephalus), no new significant enlargement of the ventricles, and persistent regression of the hemorrhage. The treatment strategy focused on blood pressure management, dexamethasone tapering, nimodipine administration for cerebral vasospasm prophylaxis, and early mobilization and rehabilitation.

On Day 8, the EVD was increased to 25 cm H₂O to continue the weaning process; however, since that time, CSF drainage had ceased, and the patient began experiencing a more severe headache. There was no vomiting, confusion, or change in the state of consciousness. Neurological examination was normal, with no acute deficits noted aside from a mild decrease in serum sodium. The EVD was clamped pending follow-up brain imaging and angiography the following day. To address the worsening headache, antihypertensive medications were titrated, dexamethasone was discontinued, and the analgesic regimen was optimized alongside escalated clinical monitoring.

On Day 9, the patient was experiencing progressive improvement, and the EVD was removed after an unremarkable angiography, which indicated the absence of aneurysms or vascular malformations. Aside from mild hyponatremia, laboratory parameters remained within normal limits, with effective pain control and optimized blood pressure and glycemic levels. Given the patient's ongoing clinical stability, he was transferred from the ICU to step-down care.

On Day 10, the patient remained fully alert, oriented, and neurologically stable. Pain management and volume status were sufficient, but the laboratory results demonstrated mild hyponatremia and a low albumin level, with intact kidney function. Repeat head CT was reassuring, demonstrating only a small amount of residual blood, as well as near-complete resolution of intracerebral air. Preadmission antihypertensive, lipid-lowering, antidiabetic, and analgesic regimens were continued or adjusted as clinically indicated, alongside routine DVT prophylaxis.

On Day 11, the patient was clinically and neurologically stable, with no new complaints. His examination was normal, and his laboratory parameters were unremarkable apart from hyponatremia, with a sodium level of 132 mmol/L. He was cleared for transfer to the neurosurgical ward, where he could be monitored regularly and undergo follow-up scans, given his continued clinical stability with supportive care in place.

On Day 12, the patient showed steady and ongoing improvement, with stable clinical and neurological status, and the care team planned for discharge. He and his family were carefully educated about medication scheduling, the importance of rest, and the need to monitor for important symptoms such as severe headache, vomiting, or new confusion, which would warrant immediate medical attention. Potential complications were discussed with the family, including the signs and symptoms to watch for while monitoring his recovery at home. Plans were made for follow-up with the neurosurgery team and coordination of outpatient care to facilitate his continued recovery.

CT of the head indicated diffuse SAH with hydrocephalus (Figure 1), and additional vascular imaging indicated the absence of aneurysmal or vascular malformations (Figures 2A-2B), thus confirming the diagnosis of non-aneurysmal SAH.

**Figure 1:**
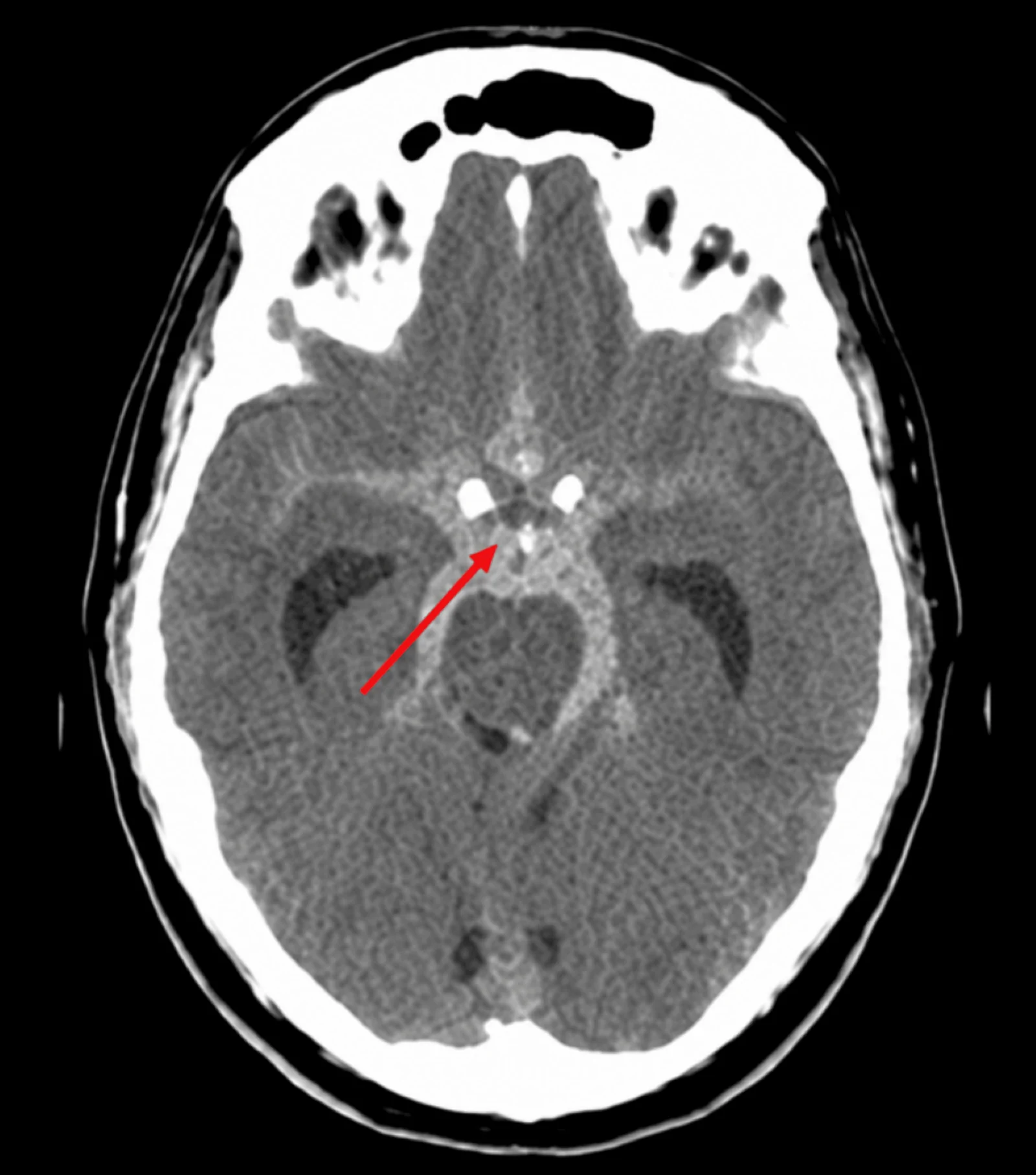
Non-contrast CT head Axial non-contrast CT of the head obtained on admission shows diffuse subarachnoid hemorrhage layering within the basal cisterns, with associated acute hydrocephalus; the arrow indicates hyperdense acute blood within the basal cisterns. CT: computed tomography

**Figure 2:**
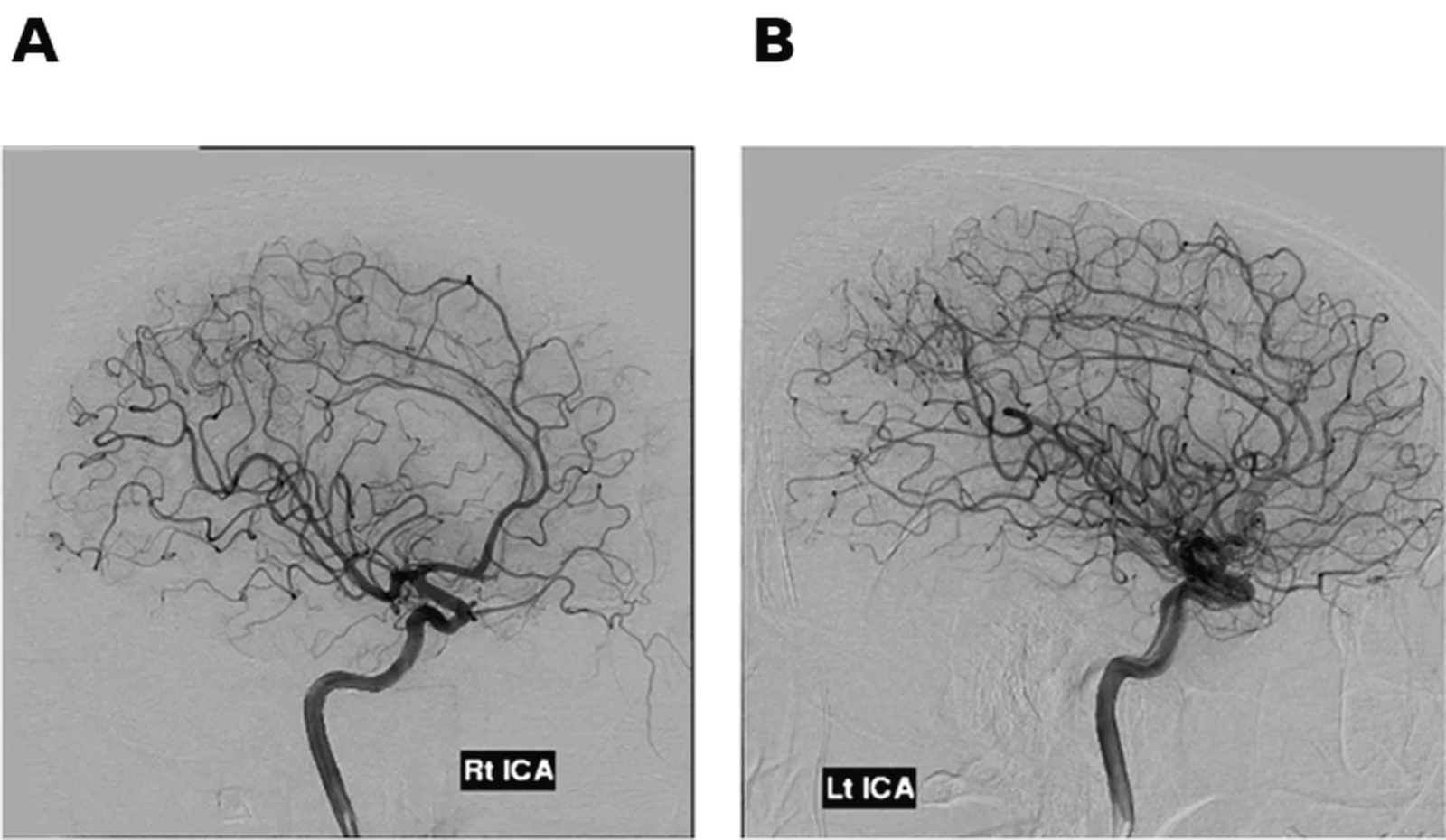
Cerebral catheter angiography A) Right internal carotid artery (ICA) angiogram; B) Left ICA angiogram. Both show normal intracranial vasculature, with no aneurysm or vascular malformation identified.

## Discussion

SAH following dental extraction

SAH can occur spontaneously after dental extraction. There are rare instances of SAH temporally associated with dental extraction, though these exist only as isolated case reports rather than in controlled studies [3,6]. This clinical episode is notable because the majority of published cases of non-aneurysmal SAH do not have a dental procedure clearly linked to the occurrence [3]. These temporal relationships observed in such rare documented instances suggest that dental work may serve as a precipitating factor, particularly in patients with vascular risk factors or underlying fragile small vessels [7].

The incidence

Non-aneurysmal SAH is responsible for 15% of cases of spontaneous SAH, and its incidence after dental extraction is unknown because it is extremely rare [5]. The overall incidence of all-cause SAH is approximately 8 per 100,000 population per year, of which only a minority are non-aneurysmal [4]. This report lacks sufficient epidemiologic evidence to support a direct causal relationship between dental procedures and the development of SAH [3].

Pathophysiology

Although its pathophysiology is not fully clear, some possible pathophysiological pathways can potentially be proposed between dental extraction and SAH.

Hemodynamic Stress

Dental manipulation or anxiety-induced sympathetic discharge can increase local or systemic blood pressure and thereby cause rupture of small, delicate vessels located in the subarachnoid space [6].

Valsalva Maneuver

Coughing, straining, or holding the breath during the procedure can temporarily raise intracranial venous pressure, which may result in rupture of a vessel, especially in patients with microvascular disease [8].

Inflammatory Cascade

Local infection, bacteremia, or inflammation caused by dental procedures is a rare cause of vascular wall weakness [9].

Patient Comorbidities

Chronic hypertension, diabetes, dyslipidemia, and renal dysfunction enhance microvascular fragility and susceptibility to bleeding [7].

Generally, the exact mechanism in non-aneurysmal SAH cases is speculative in the majority of reported cases. Rarely, rupture of small perforating vessels or venous infarction is suspected [10].

Treatment and prevention

Acute Management

CSF diversion: CSF diversion should be performed early by inserting an EVD in cases complicated by acute hydrocephalus to normalize intracranial pressure and allow neurological monitoring [4].

Supportive care in ICU: Careful monitoring of fluid, electrolyte, and metabolic status, along with secondary prevention of complications (e.g., infection, vasospasm, or hyponatremia), is crucial for recovery [4].

Nimodipine: Nimodipine serves a neuroprotective role by reducing the risk of cerebral vasospasm [4].

Multidisciplinary management: Patients who have significant comorbidities should be managed in coordination with nephrology, endocrinology, and critical care teams [4].

Prevention

Although exceptionally uncommon, prevention is based on achieving optimal management of predisposing comorbid conditions (hypertension and diabetes), proper control of blood pressure during and after the procedure, and counselling of patients regarding the low but possible risk of neurological events after dental procedures, particularly in high-risk groups [7].

## Conclusions

An extraordinarily rare circulatory event is the occurrence of non-aneurysmal SAH with the development of hydrocephalus following a dental extraction, and the incidence of such cases is limited to case reports. The case highlights the realization that seemingly mundane dental procedures, when performed in patients with predisposing comorbidities, can trigger severe neurological sequelae. A key component of patient recovery is early diagnosis and management, especially ventricular drainage to manage hydrocephalus and intensive care management. Ensuring that all vascular risk factors are properly managed and monitoring high-risk individuals are central to prevention, although the overall risk is extremely low. In conclusion, positive patient outcomes, such as that observed in the case presented, are determined by immediate, hands-on multidisciplinary action and close follow-up throughout a patient's hospital stay.
